# Autre cause de mort subite du nourrisson: à propos d'un cas clinique de syndrome du QT long congenital

**DOI:** 10.11604/pamj.2014.19.46.4703

**Published:** 2014-09-19

**Authors:** Zena Seka, Pierre Mols, Eric Gobin, William Ngatchou

**Affiliations:** 1 Clinique Sainte Elisabeth, Namur, Belgique; 2 CHU Saint Pierre, Bruxelles, Belgique; 3 Clinique Le Noirmont, Le Noirmont, Suisse

**Keywords:** Syndrome du QT long, canalopathie rythmique, mort subite, Long QT Syndrome, rhythmic channelopathy, sudden death

## Abstract

Le syndrome du QT long congénital est une maladie rythmique liée à une mutation génétique et caractérisée par un espace QT allongé sur l’électrocardiogramme, des arythmies malignes type torsade de pointe et fibrillation ventriculaire entraînant une mort subite. Les gènes impliqués dans ces mutations codent pour des sous unités des canaux ioniques responsables de l'activité électrique cardiaque. Le diagnostic est basé sur l’électrocardiogramme, une enquête familiale et l’étude génétique. Le traitement repose sur les bêtabloquants, la sympathectomie et le stimulateur cardiaque. Nous rapportons le cas d'un nourrisson de 2 ans retrouvé en état de mort apparente. Nous discutons de sa prise en charge initiale, de l'enquête familiale et de son suivi ultérieur.

## Introduction

Le syndrome du QT long congénital est une maladie rythmique liée à une mutation génétique et caractérisée par un espace QT allongé sur l’électrocardiogramme de surface. Cette pathologie représente un risque vital pour l'individu porteur, qui présente des syncopes par torsade de pointe aboutissant à de la fibrillation ventriculaire et une mort subite mais la première manifestation peut être une crise d’épilepsie [1, 2].

Les gènes impliqués dans la mutation codent pour des sous unités des canaux ioniques responsables de l'activité électrique du cœur [3]. Son incidence est estimée à 1/2000, selon certaines études [4]. Il existe actuellement quatre phénotypes cliniques [5, 6]. Le diagnostic repose sur l’électrocardiogramme de surface qui montre un allongement de l'espace QT supérieur à 440 ms associé à des anomalies de morphologiques de l'onde T, et parfois une bradycardie. Chez le sujet sain, l'espace QT est de 440 ms, cet espace est influencée par la fréquence cardiaque et le tonus neuro-végétatif, d'où la notion de l'intervalle QT corrigé adapté au rythme cardiaque. Selon la formule de Barzett, l'intervalle QT corrigé est égal au rapport entre l'intervalle QT en milliseconde divisé par la racine carrée de l'intervalle RR du battement précédent. Un espace QT corrigé supérieure à 440 ms serait associé à une forte probabilité de syndrome du QT long [7]. Au cours d'une étude, il a été démontré que 12% de patients génétiquement atteints avaient un QTC normal, inférieur à 440 ms [8].

**Figure 1:**
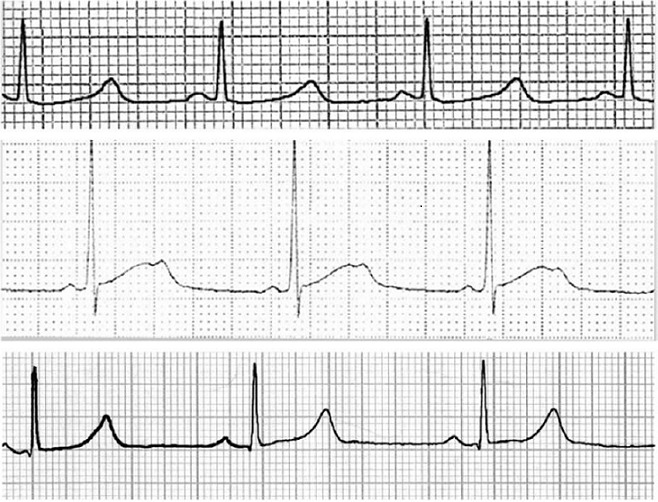
De haut en bas: un ECG typique de LQT1, avec une base élargie, en deuxième position, un tracé typique du LQT2 avec son aspect en double bosse, en dernière position vient le tracé d'un LQT3 difficile à différentier du LQT1

**Figure 2:**
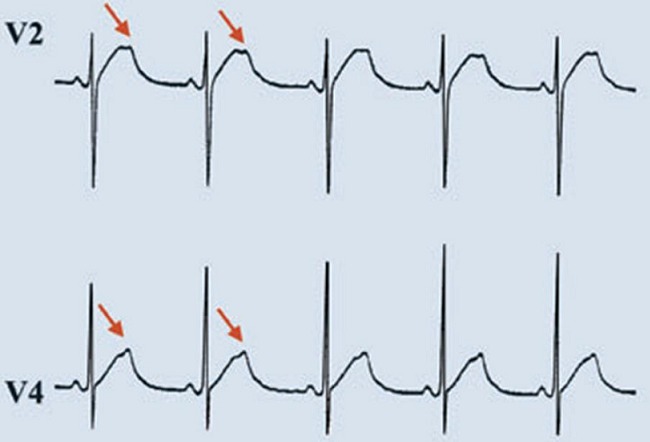
Tracé ECG montrant des ondes T en double bosse pathognomonique du LQT2 (d'après Blackwell Publishing Ltd Journal of Internal Medicine. 2005; 259: 39–47)

En effet, Il existe un chevauchement important des courbes de distribution des espaces QT chez les sujets sains et chez les sujets atteints [9]. D'autres critères comme l'analyse de la morphologie de l'onde T ont été pris en considération par l’équipe de cardiologie de l'hôpital Lariboisière à l'Assistance Publique de Paris. L'analyse morphologique de l'onde T de sujets génétiquement identifiés montre des aspects spécifiques de chacun des trois locus les plus fréquemment impliqués dans la maladie. Dans la forme LQT3 l'intervalle QTc est très allongé avec une onde T tardive et de grande amplitude [10]. Dans la forme LQT1, la morphologie de l'onde T est monophasique avec une base élargie [3] (Figure 1, 1er tracé). Dans 33% des cas, l'ECG ne permet pas une orientation différentielle entre KCNQ1 (LQT1) et SCN5A (LQT3) et dans 7% des cas de mutations dans KCNH2 (LQT2), l'ECG n'est pas spécifique [9]. Dans la forme LQT2, on retrouve un aspect de l'onde T en double bosse (Figure 1 sur le 2ème tracé, Figure 2) Compte tenu de la variation nycthémérale de l'espace QT, un holter 24h peux être réalisé dans les cas douteux. Le test d′effort peut être utile afin de documenter la non adaptation de l′intervalle QT aux variations de fréquence cardiaque [11]. L'enquête familiale s'avère utile pour la recherche d'une histoire de mort subite du nourrisson, d'accident de noyade, de syncope ou d’épilepsie.

Le test génétique est utilisé dans deux situations: (1) En cas de clinique fortement suspecte, le test génétique permet de confirmer le diagnostic. (2) Lorsque la mutation familiale est connue, elle peut être recherchée chez tous les collatéraux de cette famille par le test génétique, dans le but d'un traitement éventuel.

Un test génétique négatif chez un patient cliniquement atteint ne remet pas en cause le diagnostic.

Le traitement du syndrome du QT long congénital repose sur l’éviction des médicaments allongeant l'espace QT et la correction de désordres hydro électrolytiques. Les bêtabloquants ont pour but d′éviter les effets liés à l′augmentation du tonus adrénergique. On préfère utiliser les molécules à longue durée d'action [12]. Le Sotalol est le seul bêtabloquant contre indiqué dans le syndrome du QT long, a cause de son effet propre sur la repolarisation. La stellectomie gauche ou dénervation sympathique gauche est utilisée en cas d’échec des bêtabloquants: la technique est couramment utilisée en Italie par l’équipe de Peter Schwartz et consiste à retirer le ganglion stellaire gauche en prévention d'arythmies malignes [13]. Le Sulfate de magnésium trouve sa place en cas de torsade de pointe [14]. Le pace maker peut être indiqué lorsque la fréquence cardiaque spontanée est très lente et quand la bradycardie favorise la survenue des troubles du rythme [15]. Les inhibiteurs des canaux sodiques par les anti-arythmiques de classe 1B combinés à la mise en place d′un stimulateur cardiaque sont en cours d’évaluation [16].

## Patient et observation

Une fillette de 2 ans est trouvée en état de mort apparente par sa famille. Il s’écoule un délai de 13 minutes entre la découverte de l'enfant par sa famille et le début des manœuvres de réanimation. A l'arrivée du réanimateur qui est voisin de la famille, l'enfant est hypotonique, elle présente une cyanose labiale avec une pâleur extrême, les yeux sont grands ouverts avec présence de matières alimentaires obstruant la cavité oro-pharyngée. Il n'y a pas de respiration spontanée ni de pouls; le diagnostic de mort apparente est établi.

Les man'uvres de réanimation par dégagement des voies aériennes, massages cardiaques externes alternés avec des insufflations pulmonaires permettent au bout de 5 minutes la reprise de l'activité cardiaque, respiratoire et le retour a la conscience. L'enfant sera ultérieurement transféré dans un service d'urgences.

L'anamnèse médicale réalisée aux urgences révèle que l'enfant est porteur d'une mutation génétique responsable du syndrome du QT long congénital, elle est traitée par bêtabloquant à base de Nadolol (Corgard^®^) à raison de 3 mg/kg/j, soit 20mg, 2/j. L'enfant avait bien pris son traitement. L'examen clinique réalisé aux urgences montre un poids de 13 kg, une pression artérielle de 80/60 mmHg, la fréquence cardiaque est de 126/minute, la température est à 38,8°C.

**Figure 3:**
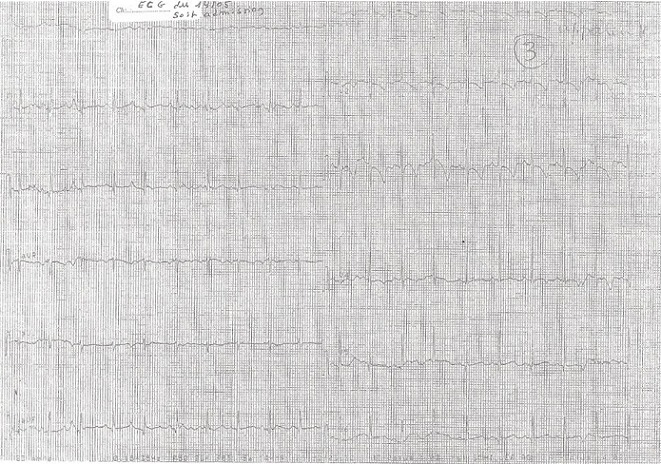
ECG à l'admission

Le bilan réalisé à son admission aux urgences montre à l’électrocardiogramme un rythme sinusal régulier, à 105/min, on note une onde T asymétrique, l'espace QT corrigé est de 450 millisecondes (Figure 3). Le bilan réalisé en cours d'hospitalisation est tout a fait rassurant et montre un ionogramme normal, l'absence de germes au niveau du sédiment urinaire, il n'y a pas de foyer pulmonaire a la radiographie du thorax, la tomodensitométrie cérébrale est normale, l’électroencéphalogramme ne montre pas de foyer comitial. L’échographie cardiaque est normale. On note une excellente évolution durant l'hospitalisation, le nourrisson ne présente pas de séquelles neurologiques, ni de récidive d'arythmies.

Un diagnostic de mort subite dans un contexte de syndrome du QT Long est posé et la dose de Nadolol majorée à 25 mg deux prises par jour. En cas de récidive, la pose d'un défibrillateur est envisagée.

Dix mois après l'incident cardiaque, l'enfant a été pris en charge par une équipe d'aide médicale urgente pour crise d’épilepsie inaugural et l’électroencéphalogramme réalisé aux urgences ne montre pas de foyer comitial. Un traitement à base d'acide valproique fut instauré. Actuellement, l'enfant est scolarisée dans une institution spécialisée, elle présente un retard scolaire et est toujours traitée par bêtabloquant et antiépileptique.

**Figure 4:**
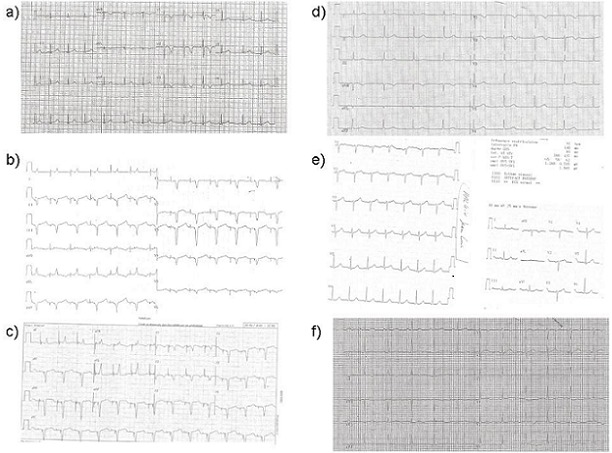
Enquête familiale: a) Tracé ECG de sa petite sœur, elle aussi atteinte et traitée par bêtabloquant, b) ECG de sa maman atteinte elle aussi, c) ECG de sa tante atteinte elle aussi, d) sa tante Stéphanie est également atteinte, e) grand-père maternel porteur de mutation mais qui n'est pas traité, f) grand-mère maternelle porteuse de mutation et qui n'est pas traitée

L'enquête familiale (Figure 4) faite aux urgences révèle une mère porteuse d'une double mutation génétique au niveau du gène HERG et du gène KCNQ1. Elle est traitée par bêtabloquant et porte un stimulateur cardiaque. La sœur du nourrisson porte également une des mutations de la mère: elle est positive pour la mutation au niveau du gène HERG, négative pour le gène KCNQ1, elle est traitée par bêtabloquant. La tante maternelle du nourrisson est porteuse d'une double mutation. Elle a bénéficié d'une sympathectomie urgente à Milan, elle porte un défibrillateur, et elle est traitée par bêtabloquant et vérapamil. Les 2 grands-parents maternels sont génétiquement positifs mais ne sont pas traités.

## Discussion

Le syndrome du QT Long congénital est une affection rare mais avec de lourdes conséquences si la maladie n'a pas été détectée et traitée a temps. L'intérêt de notre cas est de montrer que les patients même traités ne sont pas à l'abri d'une arythmie fatale. De même notre cas révèle la complexité de la prise en charge.

Priori SG et al ont en effet montré que 10% de patients porteurs de la mutation LQT1 connaissent un accident cardiaque grave malgré un traitement sous bêtabloquants bien conduit [12].

En cas d’échec des bêtabloquants, la sympathectomie représente un traitement complémentaire mais elle aussi ne protège pas le patient à 100%. La tante de notre patient a bénéficié d'un tel traitement sans succès justifiant l'implantation d'un défibrillateur. L'implantation de ce dernier étant recommandé dans la prévention secondaire de la mort subite [16, 17] chez ces patients.

L'enquête familiale montre que notre patiente et sa tante ont présenté des crises d’épilepsies; ces dernières pouvant être la première manifestation symptomatique. Certains auteurs soulignent l'association QT long et épilepsie comme étant deux entités étroitement liées; les crises d’épilepsie ne sont pas en rapport avec les répercussions hémodynamiques secondaire à l'arythmie. Chez certains auteurs, ces affirmations sont étayées par l'existence au niveau du SNC de la souris d'un gène responsable de l'activité épileptique et qui code pour les canaux ioniques responsables du syndrome du QT.

Il est recommandé de traiter les patients symptomatiques avec un test génétique positif ou non.

Les patients asymptomatiques avec un test génétique positif doivent être traités par bêtabloquant dans le but de les protéger de la mort subite qui constitue parfois la première manifestation. Dans notre ce cas qui nous préoccupe, les grands-parents de l'enfant sont génétiquement positifs mais ne sont pas traités, contrairement aux recommandations de littérature. Le traitement des collatéraux asymptomatiques avec test génétique négatif reste débattu.

## Conclusion

Notre cas clinique montre la complexité de la prise en charge de cette pathologie. Dans tous les cas, le clinicien doit rester proactif et assurer un suivi étroit des patients.

## References

[1] Omichi Chikaya, Momose Yoshio, Kitahara Shigemi (2010). Congenital long QT syndrome presenting with a history of epilepsy: misdiagnosis or relationship between channelopathies of the heart and brain?. Epilepsia..

[2] Johnson JN, Hofman N, Haglund CM, Cascino GD, Wilde AA, Ackerman MJ (2009). Identification of a possible pathogenic link between congenital long QT syndrome and epilepsy. Neurology..

[3] Schwartz PJ (2006). The congenital long QT syndromes from genotype to phenotype: clinical implications. J Intern Med..

[4] Schwartz PJ, Stramba-Badiale M, Crotti L, Pedrazzini M, Besana A, Bosi G, Gabbarini F, Goulene K, Insolia R, Mannarino S, Mosca F, Nespoli L, Rimini A, rosati E, Salice P, Spazzolini C (2009). Prevalence of the congenital long-QT syndrome. Circulation..

[5] Hanazono N, Tanaka R (1975). QT prolongation and syncopal attacks: A case of the Romano-Ward syndrome. Jpn Heart J..

[6] Tranebjaerg L, Bathen J, Tyson J, Bitner-Glindzicz M (1999). Jervell and Lange-Nielsen syndrome: a Norwegian perspective. Am J Med Genet..

[7] Schwartz PJ, Moss AJ, Vincent GM, Crampton RS (1993). Diagnostic criteria for the long QT syndrome: An update. Circulation..

[8] Vincent GM, Timothy KW, Leppert M, Keating M (1992). The spectrum of symptoms and QT intervals in carriers of gene for the long QT syndrome. N Engl J Med..

[9] Zhang L, Timothy KW, Vincent GM, Lehmann MH, Fox J, Giuli LC, Shen J, Splawski I, Priori SG, Compton SJ, Yanowitz F, Benhorin J, Moss AJ, Schwartz PJ, Robinson JL, Wang Q, Zareba W, Keating MT, Towbin JA, Napolitano C, Medina A (2000). Spectrum of ST-T wave patterns and repolarization parameters in congenital long QT syndrome ECG findings identify genotypes. Circulation..

[10] Moss AJ, Zareba W, Benhorin J, Locati EH, Hall WJ, Robinson JL, Schwartz PJ, Towbin JA, Vincent GM, Lehmann MH (1995). ECGT-wave patterns in genetically distinct forms of hereditary long QT syndrome. Circulation..

[11] Viskin S, Postema PG, Bhuiyan ZA, Rosso R, Kalman JM, Vohra JK, Guevara-Valdivia ME, Marquez MF, Kogan E, Belhassen B, Glikson M, Strasberg B, Antzelevitch C, Wilde AA (2010). The response of the QT interval to the brief tachycardia provoked by standing: a bedside test for diagnosing long QT syndrome. J Am Coll Cardiol..

[12] Priori SG, Napolitano C, Schwartz PJ, Grillo M, Bloise R, Ronchetti E, Moncalvo C, Tulipani C, Veia A, Bottelli G, Nastoli J (2004). Association of long QT syndrome loci and cardiac events among patients treated with beta-blockers. JAMA..

[13] Schwartz PJ, Priori SG, Cerrone M, Spazzolini C, Odero A, Napolitano C, Bloise R, De Ferrari GM, Klersy C, Moss AJ, Zareba W, Robinson JL, Hall WJ, Brink PA, Toivonen L, Epstein AE, Li C, Hu D (2004). Left cardiac sympathetic denervation in the management of high-risk patients affected by the long-QT syndrome. Circulation..

[14] Hasegawa J, Takami T, Kaneda T, Yamane W, Hoshio A, Igawa O, Fujimoto Y, Kotake H, Mashiba H (1991). Treatment of torsade de pointes with intravenous magnesium in idiopathic long QT syndrome. Jpn Circ J..

[15] Eldar M, Griffin JC, Abbott JA, Benditt D, Bhandari A, Herre JM, Benson DW, Scheinman MM (1987). Permanent cardiac pacing in patients with the long QT syndrome. J Am Coll Cardiol..

[16] Schwartz PJ, Prior SG, Locati EH, Napolitano C, Cantu F, Towbin JA, Keating MT, Hammoude H, Brown AM, Chen LS (1995). Long QT syndrome patients with mutations of the SCN5A and HERG genes have differential responses to Na+ channel blockade and to increases in heart rate: Implications for gene-specific therapy. Circulation..

[17] Horner JM, Kinoshita M, Webster TL, Haglund CM, Friedman PA, Ackerman MJ (2010). Implantable cardioverter defibrillator therapy for congenital long QT syndrome: a single center experience. Heart Rhythm..

